# Seroepidemiological study of ovine toxoplasmosis in East and West Shewa Zones of Oromia Regional State, Central Ethiopia

**DOI:** 10.1186/1746-6148-9-117

**Published:** 2013-06-15

**Authors:** Endrias Zewdu Gebremedhin, Abebe Agonafir, Tesfaye Sisay Tessema, Getachew Tilahun, Girmay Medhin, Maria Vitale, Vincenzo Di Marco, Eric Cox, Jozef Vercruysse, Pierre Dorny

**Affiliations:** 1 Department of Veterinary Laboratory Technology, Faculty of Agriculture and Veterinary Sciences, Ambo University, P O Box 19, Ambo, Ethiopia; 2 Faculty of Veterinary Medicine, Jijiga University, Jijiga, Ethiopia; 3 Department of Microbiology, Epidemiology and Public Health, College of Veterinary Medicine and Agriculture, Addis Ababa University, Debre-Zeit, Ethiopia; 4 Addis Ababa University, Aklilu Lemma Institute of Pathobiology, P O Box 1176, Addis Ababa, Ethiopia; 5 Italian National Reference Centre for Toxoplasmosis at Istituto Zooprofilattico Sperimentale Della Sicilia A, Mirri, Italy; 6 Faculty of Veterinary Medicine, Department of Virology, Parasitology and Immunology, Ghent University, Salisburylaan 133, Merelbeke B-9820, Belgium; 7 Department of Biomedical Sciences, Institute of Tropical Medicine, Antwerp B-2000, Belgium

**Keywords:** *Toxoplasma gondii*, Sheep, Central Ethiopia, ELISA, Seroprevalence

## Abstract

**Background:**

Toxoplasmosis is a globally distributed zoonosis. Consumption of raw or undercooked meat, which is among the main risk factors for acquiring human infection, is a popular tradition in Ethiopia. However, studies on toxoplasmosis in food animals used for human consumption in Ethiopia are very scarce. Thus, the objectives of the present study were to estimate the seroprevalence and the risk factors of *T. gondii* infection in sheep in Ambo, Ada’a-Liben and Fentale districts of Central Ethiopia. Sera from 1130 sheep were analyzed for *Toxoplasma gondii* specific IgG antibodies using an indirect enzyme linked immunosorbent assay (ELISA) with the P30 antigen. A questionnaire was administered to assess potential risk factors for *T. gondii* seropositivity. Association of seroprevalence with potential risk factors related to altitude, host and farm characteristics were analyzed by univariable and multivariable logistic regression.

**Results:**

Overall flock and animal level seroprevalences were 70.48% (160/227; 95% CI: 64.51, 76.46) and 31.59% (357/1130; 95% CI: 28.88, 34.31), respectively. The multivariable logistic regression model indicated that the probability of acquiring *T. gondii* was higher in sheep from highland (2300 – 3200 meters above sea level) [Odds ratio (OR) = 4.11, 95% confidence interval (CI): 2.65, 6.36; P < 0.001] and midland (OR = 4.54, 95% CI: 2.76, 7.49; P < 0.001) than from lowland (<1500 meters above sea level), in females than in males (OR = 1.60, 95% CI: 1.04, 2.43, P = 0.033), in adult than in young animals (OR = 2.93, 95% CI: 1.97, 4.35, P < 0.001), in small than in large flocks (OR = 3.34, 95% CI: 1.26, 8.86, P = 0.016), and in sheep that were given tap water (OR = 4.07, 95% CI: 1.07, 15.42, P = 0.039) and river water (OR = 4.18, 95% CI: 1.54, 11.35, P = 0.005) than in those that drunk water from mixed sources (i.e., river, well, lake and pond).

**Conclusions:**

The high flock and animal level seroprevalence of toxoplasmosis in sheep is a good marker of the potential risk for human infections. Altitude, sex, age, flock size and source of water were identified as important risk factors to acquire the infection. Public education and awareness training are imperative in order to alleviate the danger posed to consumers. Further detailed studies to assess the impact of infections are warranted.

## Background

Sheep are important for mutton, wool and milk production throughout the world [1]. There are approximately 26.1 million sheep in Ethiopia [2]. Mutton is the most popular source of animal proteins in Ethiopia as well as in the importing countries (Arabian countries). It is estimated that 1,078,000 sheep are consumed in Ethiopia while 700,000 sheep are exported annually [3].

Toxoplasmosis is one of the most globally widespread zoonoses with considerable health and economic impacts. The disease is recognized as an emerging food-borne parasitic disease [4]. In some countries *T. gondii* is among the highest ranking foodborne pathogens causing high disease burden both at individual and population levels [5].

Domestic cats and wild felids play a crucial role in the epidemiology of toxoplasmosis as the definitive hosts, through the shedding of millions of oocysts when infected [6,7]. Human beings and other warm-blooded animals become infected primarily by ingesting food or water contaminated with sporulated oocysts or by ingesting meat that contain tissue cysts of *T. gondii*[6,8-11]. Infection of pregnant women may result in abortion or congenital infection that may cause hydrocephalus, intracranial calcification and retinochoroiditis [10]. In addition, recent studies have indicated that toxoplasmosis is a plausible risk factor for personality shifts and increased likelihood of reduced intelligence or schizophrenia [12]. There is also a very high correlation between latent toxoplasmosis and car accidents [13,14]. Recently, highly virulent genetically atypical strains of *T. gondii* have been incriminated with pneumonia, even in immunocompetent individuals [15].

Toxoplasmosis leads to great economic losses in sheep and goats by causing embryonic death and resorption, fetal death and mummification, abortion, stillbirth, neonatal death and reduced flock milk production and infertility [16-18]. Antibodies to *T. gondii* have been found in sheep worldwide [6]. Seropositive small ruminants can be assumed to harbor tissue cysts in their meat [19,20] thereby endangering the consumers of mutton.

*Toxoplasma gondii* infection of sheep and goats has been reported from various African countries [21-23]. Previous studies in Ethiopia have shown that seroprevalence of anti-*T. gondii* antibodies in sheep ranges from 11.9% to 56% [24-26]. However, the epidemiology of toxoplasmosis in food animals and humans is largely unknown in Ethiopia. The objectives of the present study were to estimate the seroprevalence and the risk factors of *T. gondii* infection in sheep in Ambo, Ada’a-Liben and Fentale districts of Central Ethiopia.

## Methods

### Description of the study districts and population

The study was conducted in three districts of Oromia regional state, Central Ethiopia. The study areas are separated from each other by 150 to 289 kms and represent the highland (≥2300 meters above sea level [mas]), midland (1500 – 2300 masl) and lowland (< 1500 masl) agro-ecologies of the region.

Ambo district (longitude 37° 32' to 38° 3′ E and latitude 8° 47′ to 9° 20′ N) is located in Western Shewa zone of the Oromia Regional State. The altitude within the district ranges from 1400 to 3045 masl. Majority of the places in Ambo district are high land. The annual rainfall and temperature range from 800 – 1000 mm and 15°C – 29°C, respectively. The mean temperature is 18.6°C.

Ada’a-Liben (longitude 38° 58′E to 39° 22′E and latitude 08° 22′N to 8° 56′ N) and Fentale Districts (longitude 39.93°E to 39° 56′0″E and latitude 8.975° N to 8.58′30″ N) are located in East Shewa zone at a distance of 45 kms and 190 kms from Addis Ababa, respectively. The altitude of Ada’a-Liben and Fentale districts range from 1500 to over 2000 and 955 to 2007 masl, respectively. The vast majority of Fentale is lowland (except at a place called mount Fentale where altitude reaches 2007 m) while that of Ada’a-Liben district is midland). The average rainfall and temperature of Ada’a-Liben and Fentale districts are about 839 mm and 553 mm and 7.9°C to 28°C and 29 to 38°C, respectively. Fentale district has an arid to semi-arid climate and the production system is predominantly pastoral and agro-pastoral. Sedentary farming dominated by extensive type of management system is a feature of the highlands and midlands of Ambo and Ada’a-Liben districts. However, semi-intensive farming is practiced in some urban and peri-urban areas.

Afar, Arsi-Bale and Horro breeds of sheep predominate in Central Ethiopia. Sheep are kept for mutton production in most parts of the country; however, pastoralists in Fentale district also use sheep for milk production. In this study, sheep of both sexes above six months old were included. Sheep aged ≤ 1 year are considered as young while those above 1 year are considered as adult.

### Study design and sample size

A cross-sectional study with a two-stage cluster sampling design was carried out from November 2010 to April 2011 in order to estimate the flock and individual animal level sero-prevalence. Districts, peasant associations and flocks were conveniently chosen. An expected animal level prevalence of 54.7% [24] and 5% absolute precision were used to calculate the required sample size followed by a three times inflation. This is because of the absence of variance data between clusters and our interest of having a more precise estimate [27,28]. The required sample size (n = 1130) was allocated to each district proportionally based on their sheep population. The number of sheep flocks (n = 227) [Table 1] to visit was determined by dividing the total sample size (n = 1130) with the number of sheep to be sampled within each flock (i.e. 5). For sample size calculation the average number of sheep per household suitable for sampling (≥ 6 months) was assumed to be five. In a flock with ≤ 5 sheep, all were sampled. However, from a flock comprised of more than five sheep a random sample of 5 animals was selected.

**Table 1:** Summary of peasant associations and flocks sampled from Ambo, Fentale and Ada’a-Liben districts

**Districts**	**PA’s**		**Flocks sampled**		**Flocks (sampled/total)**
	**Total**	**Sampled**	**Extensive system**	**Semi-intensive system**	
Ambo	34	10	70	8	78/6079
Ada’a-Liben	22	6	12	36	48/3676
Fentale	18	7	98	3	101/2161
Total	74	23	180	47	227/11916

Total number of peasant associations (PA’s) in study districts, number of PA’s sampled, and number of flocks sampled from extensive and semi-intensive system of management along with total number of flocks sampled.

### Blood collection and serum separation

Approximately 5 ml of whole blood samples were collected by venipuncture from the jugular vein using disposable plain vacutainer tubes and needles (BD Vacutainer Systems, Plymouth, UK). The blood samples were allowed to clot and then centrifuged at 4000 rpm for 5 minutes. Centrifugation took place in the field, i.e., during the evening in the town of each district. The serum was collected into 1.5 ml Eppendorf tubes (Eppendorf-AG, Hamburg, Germany) and transported to the School of Veterinary Medicine, Debre-Zeit, using an ice box and stored at −20°C until serologically tested.

### Questionnaire survey

A close-ended questionnaire was developed and filled in at flock or household level by interviewing flock owners or herders during sampling in order to assess potential risk factors for toxoplasmosis. Those included flock size, presence of cats in the households, presence of wild felids in the area, type of management (extensive: free ranging without supplementary feed; semi-intensive: supplementary feed provided), source of water and grazing/browsing place. Information about altitude and farming system of sampling sites was also collected. At animal level information about breed, sex and age was collected. Animal age determination was made based on dentition [29] and herders’ information.

### ELISA

All the collected serum samples were tested for the presence of IgG antibodies against *T. gondii* following the protocol of the manufacturer of the indirect enzyme linked immunosorbent assay (P-30 ELISA) kit (ID VET Innovative Diagnostic, ID Screen®, Montpellier, France), which is coated with the P30 surface antigen of the organism.

### Data management and analysis

The data generated were stored in Microsoft excel spreadsheet (Microsoft Corporation) and analyzed using STATA version 11.0 for MA Windows (Stata Corp. College Station, USA). Univariable logistic regression analysis was performed, and odds ratios (OR) and 95% confidence intervals (CI 95%) were used to quantify the association between risk factors and *T. gondii* infection. Continuous data were collected from the field for age and flock size. These variables were categorized during data analysis. The categories of the variables were as follows. Altitude (lowland, mid land, high land), sex (male vs. female), age (young vs. adult), flock size (large vs. small taking 50 as a cut-off), management type (extensive vs. semi-extensive), residential place (urban vs. rural), source of drinking water (stagnant, river, tap, mixed), grazing land (plain, mountainous, mixed), presence of cats (present vs. absent) and presence of wild felids (yes vs. no). Hence, all variables were handled as categorical variables. Collinear variables with lpl >0.5 were identified using a collinearity matrix. One of the recommended methods of handling collinear variables is to eliminate less plausible ones. Hence, elimination of one of the collinear variable was decided based on biological ground to explain the disease better. Non-collinear variables that presented P-value of < 0.15 in univariable analysis were entered in the multivariable regression model. During the analysis the clustering nature of the outcome within flock was considered by including flock as a clustering variable. This enabled us to use clustered sandwich estimator i.e., robust standard error rather than the standard error of the parameters estimated using maximum likelihood method. Variables with more than two categories were transformed into indicator (dummy) variables. Potential risk factors included in the model were selected based on the existing literature. Flocks containing at least one seropositive animal were considered positive. After obtaining final model interaction of pre-specified factors (i.e. age and sex, flock size and cat ownership, source of water and cat ownership, flock size and source of water, flock size and management, source of water and management) was assessed. The model was constructed by backward stepwise exclusion method. The variable ‘presence of cat’ was purposively allowed to join the variables used for fitting best model due to its biological importance, while the variables ‘grazing land, presence of wild felids and residential place’ were excluded due to a high p-value. Finally, the model fitness was assessed by Hosmer–Lemeshow goodness-of-fit test [28]. The reliability of the fitted model was further evaluated using receiver operating characteristic curve (ROC). The 95% confidence level was used and results were considered significant at P ≤ 0.05.

### Ethical issues

This research project was reviewed and approved by the ethical committee for animal experimentation of the College of Veterinary Medicine and Agriculture, Addis Ababa University, Ethiopia.

## Results

### Overall seroprevalence

Out of 227 flocks tested 160 (70.48%; 95%: CI: 64.51, 76.46) flocks were positive. Fifty-five (24.23%), 50 (22.03%), 30 (13.22%), 14 (6.17%) and 11 (4.85%) flocks of sheep had one, two, three, four and five seropositive animals, respectively. The overall animal level seroprevalence of *T. gondii* infection was 31.59% (357/1130; 95% CI: 28.88, 34.31). Highest animal level seroprevalence was recorded in Ambo district (183/387, 47.29%), followed by Ada’a-Liben (106/234, 45.30%) and Fentale (68/509, 13.4%). Similarly, flock seroprevalence was high in Ambo (70/78, 89.74%) and Ada’a-Liben districts (44/48, 91.67%) as compared to Fentale district (46/101, 45.54%) [Table 2].

**Table 2:** **Animal and flock level seroprevalence of *T. gondii *infection in Ambo, Fentale and Ada’a-Liben districts**

**Study districts**	**Animal level seroprevalence**			**Flock level seroprevalence**		
	**Tested**	**%**	**95% CI [Lower–upper]**	**Tested**	***%***	**95% CI [Lower–upper]**
Ambo	387	47.29	42.30, 52.27	78	89.74	82.93, 96.56
Fentale	509	13.36	10.40, 16.32	101	45.54	35.73, 55.36
Ada’a-Liben	234	45.30	38.90, 51.70	48	91.67	83.72, 99.61
Total	1130	31.59	28.88, 34.31	227	70.48	64.51, 76.46

Number of sheep and flocks tested and results of animal and flock level seroprevalence of *T. gondii* infection from each study districts along with the 95% confidence interval (95% CI) of the estimate.

### Risk factor analysis

During the statistical analysis, for all the risk factors, the first level of each independent variable (the category of a risk factor with lowest prevalence) was used as a reference category. Animal level seroprevalence of *T. gondii* was significantly different (P < 0.001) between the study altitudes. Seroprevalence was 4.30 times higher in midland and 3.97 times higher in highland compared to the lowland (Table 3).

**Table 3:** **Univariable logistic regression analysis of potential risk factors for *T. gondii *seropositivity in study districts**

**Risk factors category**		**N**^**a**^	**Prevalence (%)**	**Crude OR (95% CI)**	**P-value**
Altitude	Low land	509	13.36	1.00	
	Mid land	346	46.24	5.58 (3.79, 8.22)	<0.001
	High land	275	46.91	5.73 (3.72, 8.82)	<0.001
Breed	Afar	506	13.44	1.00	
	Arsi Bale	242	44.81	5.23 (3.42, 8.00)	<0.001
	Horro	383	47.26	5.77 (3.89, 8.56)	<0.001
Sex	Male	211	19.43	1.00	
	Female	919	34.39	2.17 (1.49, 3.16)	<0.001
Age	Young	235	17.02	1.00	
	Adult	895	35.42	2.67 (1.86, 3.85)	<0.001
Flock Size	Large (n ≥ 50)	100	4.00	1.00	
	Small (n < 50)	1030	34.17	12.51(4.93, 31.78)	<0.001
Management type	Extensive	905	28.40	1.00	
	SI	225	44.44	2.02 (1.35, 3.01)	0.001
Residential place	Rural	853	30.01	1.00	
	Urban	277	36.46	1.34 (0.92, 1.95)	0.129
Source of water	Mixed^b^	81	6.17	1.00	
	Stagnant^c^	167	32.34	7.26 (2.42, 21.84)	<0.001
	River	825	33.33	7.6 (2.81, 20.57)	<0.001
	Tap	57	40.35	10.28 (2.96, 35.71)	<0.001
Grazing land	Mixed	353	22.38	1.00	
	Plain area	585	32.14	1.64 (1.06, 2.54)	0.026
	Mountainous	192	46.88	3.06 (1.82, 5.15)	<0.001
Presence of cats	No	539	27.64	1.00	
	Yes	591	35.19	1.42 (1.00, 2.02)	0.051
Presence of wild felids	Yes	804	27.99	1.00	
	No	326	40.49	1.75 (1.23, 2.48)	0.002
Farming system	Pastoral	195	7.18	1.00	
	Agro-pastoral	310	17.42	2.73 (1.40, 5.33)	0.003
	Sedentary	625	46.24	11.12 (5.99, 20.64)	<0.001

^a ^Total number of sheep tested, ^b^ mixed = river, well, lake, pond; Stagnant ^c^ = pond, well, lake.

Abbreviation: *SI* semi-intensive.

Factors associated with *T. gondii* seropositivity in study districts with their frequency (N), % Prevalence, crude odds ratio (cOR) and corresponding 95% confidence interval (95% CI) in the univariable logistic regression analysis (using 1130 sheep sera from 3 districts and 227 flocks).

Univariable logistic regression analysis showed that the risk of infection in highland (OR = 5.73, 95% CI: 3.72, 8.82) and midland (OR = 5.58, 95% CI: 3.79, 8.21) was significantly higher than in lowland. *Toxoplasma gondii* infection in females (34.39%) was 2.17 times greater than in males (19.43%). Similarly, the likelihood of *T. gondii* infection in adult sheep (> 1 year age) (35.42%) was 2.67 times more than in young sheep (17.02%) [P < 0.001]. Uni-variable logistic regression analysis also showed that there was a significant association (P ≤ 0.05) between seropositivity and farming system, flock size, management type, grazing land, source of drinking water and presence of wild felids in the area. Residential place of sheep and presence of cats were not significantly associated with *T. gondii* seropositivity (P > 0.05) [Table 3]. There was no significant interaction between: age and sex, flock size and presence of cats, source of water and presence of cats, flock size and source of water and flock size and management type. However, significant interaction was observed between source of water and management type (P ≤ 0.05). The following variables were included in the multivariable model: altitude, sex, age, flock size, management type, residential place, source of drinking water, grazing land, presence of cats and presence of wild felids. Farming system and breed were not entered to the final model as they were collinear with each other as well as with altitude. The final multivariable logistic regression model of risk factors analysis revealed that altitude, sex, age, flock size, source of water and management type*source of water had a significant association with *T. gondii* seropositivity and hence are independent predictors (P ≤ 0.05) [Table 4]. In order to assess the contribution of collinear variables excluded from the final model (breed and farming system) separate analysis was done by removing altitude from the final model. The result indicated that the likelihood of *T. gondii* infection was 9.63 (95% CI: 5.00, 18.58; P < 0.001) and 3.31 (95% CI: 1.67, 6.55; P = 0.001) times higher in sedentary and agropastoral farming systems, respectively, when compared with pastoral farming system. Horro (OR = 4.18, 95% CI: 2.79, 6.28; P < 0.001) and Arsi-Bale (OR = 3.15, 95% CI: 1.59, 6.22; P = 0.001) breeds of sheep were more likely to give a seropositive result than the Afar breed. Assessment of model fitness to the observed data indicated that there was insignificant difference between the observed and predicted values. Hosmer-Lemeshow Chi-square (*χ*^2^) = 4.03, P = 0.779. Moreover, the sensitivity (60.5%), specificity (76.2%) and ROC analysis (area under the curve = 0.756) suggest also that the model fitted the data well (Table 4).

**Table 4:** **Multivariable logistic regression analysis of predictors of *T. gondii *infection in sheep of study districts**

**Risk factors category**		**Adjusted OR (95% CI)**	**P-value**
Altitude	Low land	1.00	-
	Mid land	4.54 (2.76, 7.49)	<0.001
	High land	4.11 (2.65, 6.36)	<0.001
Sex	Male	1.00	-
	Female	1.60 (1.04, 2.43)	0.033
Age	Young	1.00	-
	Adult	2.93 (1.97, 4.35)	<0.001
Flock Size	Large (n ≥ 50)	1.00	-
	Small (n < 50)	3.34 (1.26, 8.86)	0.016
Management type	Extensive	1.00	-
	SI	2.88 (0.96, 8.69)	0.060
Source of water	Mixed^b^	1.00	-
	Stagnant^c^	2.34 (0.69, 7.94)	0.174
	River	4.18 (1.54, 11.35)	0.005
	Tap	4.07 (1.07, 15.42)	0.039
Presence of cats	No	1.00	-
	Yes	1.11 (0.77, 1.60)	0.578
Management type*source of water	SI* Stagnant^c^	1.23 (0.30, 5.09)	0.772
	SI*river	3.64 (1.16, 11.43)	0.027
	SI*tap	2.12 (0.33, 13.42)	0.425

^b^ mixed = river, well, lake, pond; Stagnant ^c^ = pond, well, lake; * indicate interaction

Factors associated with *T. gondii* seropositivity in study districts with their frequency (N), % Prevalence, adjusted odds ratio (aOR) and corresponding 95% confidence interval (95% CI) in the final multivariable logistic regression model (using 1130 sheep sera from 3 districts and 227 flocks); Hosmer-Lemshow *χ*^2 ^= 4.03, P =0.779.

Although no population estimates of Ethiopian cats are available, the present study demonstrated that cats are favorite animals kept by 118 of 227 studied households (52.0%), mainly to control rodents around the home and in the grain stores.

## Discussion

### Overall seroprevalence

The present study reported an overall seroprevalence of 70.48% (160/227) at flock and 31.59% (357/1130) at animal level. Our results are within the range of the prevalence estimated in previous studies in Ethiopia, which ranged from 11.9% in Central Ethiopia [26] and 52.6% in Nazareth [24]. The great variation in the seroprevalence of *T. gondii* infection is likely to be the result of the geographic area, the farm management and the general hygienic conditions of the farms, and of technical issues such as, the serological method used, the cut off values and the sample size [30]. ELISA that uses P30 antigen, expressed exclusively by tachyzoites (fast multiplying form of the parasite) was used in the present study. Similar commercial ELISA has been previously used for diagnosis of *T. gondii* infection in sheep and goats with reliable results [31-33].

### Risk factors

This study showed that sheep from the highlands and midland areas of Central Ethiopia are at significantly higher risk of *T. gondii* infection than those from the lowland (P < 0.001). This variation can be explained by the difference of environmental temperature and moisture in these areas. The Ethiopian tropical climate is subjected to wide topographic-induced variations [34]. The influence of the environment on the epidemiology of toxoplasmosis has been well documented [11,35]. The lowest prevalence (13.36%) was recorded in lowland area, which is characterized by a hot and arid climate compared to the tepid to cool sub moist agro-ecology in the midland and the moist agro climate in the highlands of the study area. Highland and midland areas receive more rainfall; evaporation is relatively less and those areas have more forest canopies or vegetation. The resulting humidity is favorable to a higher chance of oocyst survival in the environment and infectivity to sheep, thereby contributing to the higher seroprevalence. It is well known that a dry climate has an adverse effect on the persistence and dissemination of oocysts of *T. gondii*[6,36]. Furthermore, it is worth mentioning, that as compared to lowland, human settlements and cat ownership are higher in mid and highland. Many farmers in the mid and the highlands practice mixed crop-livestock farming. Grazing land is limited and some farmers’ supplement sheep with grains or commercial concentrate feeds that is often protected from rodent attack by keeping cats around. Similar to our findings, Kamani et al. [21] also reported in Nigeria that a milder climate with higher rainfall and relative humidity favors a higher seroprevalence as compared to arid Sahel northern zones. However, a study from Mexico found that prevalence was higher at low altitudes [37].

Significantly higher seroprevalence in adult (35.42%) compared to young sheep (17.02%) is consistent with earlier studies [6,19,38-42] and is the result of higher likelihood of ingestion of oocysts with increasing age.

The higher seroprevalence in female sheep (34.39%) as compared to males (19.43%) is significant and might be attributed to the management system in that ewes are retained in the farm for longer periods for breeding purpose than males. Few rams are retained for mating while the majority are culled and sold for cash purpose. The hormonal difference in relation to stress of lactation and pregnancy leading to immunosuppression may also increase susceptibility to toxoplasmosis in females [43]. Teshale et al. [40] and Negash et al. [24] also reported higher seroprevalence in female sheep in Ethiopia.

*Toxoplasma gondii* seropositivity in small flocks (34.17%) was significantly higher than in large flocks (4.0%). This result could be ascribed to the fact that small flocks are often repeatedly tethered or allowed to graze on a small area close to the farm and households where domestic cats have an easy access and may contaminate the pasture and feed reserves (roughage and concentrate) around the homestead. On the other hand, large flocks are commonly found in pastoral areas where animals spend more time grazing over a wide area away from the farm and the home. We cannot exclude other sources of infection and there could also be risk factors independent of flock size that might have been distributed over fewer animals [44].

The higher seroprevalence (33.33%) in sheep that drunk river water is expected and suggest higher chance of contamination by oocysts while the higher seroprevalence (40.35%) in sheep that were given water from the tap than those drinking from both river and stagnant water bodies (6.17%) is puzzling. This may suggest that surrounding felid populations are contaminating the source of tap water with oocysts, and water purification and chlorination processes are either ineffective against oocysts or non-existent [11,45]. It could also be possible that some unknown confounding factors such as the poor sanitation of watering troughs can influence the probability of *T. gondii* seropositivity.

The presence of cats is crucial in the life cycle of *T. gondii* and is significantly associated with increased seroprevalence in many reports [6,7]. In our study the presence of cats was not an independent predictor of toxoplasmosis in the final model. We expect that in those households reporting absence of cats at their home, stray and wild cats from neighboring areas might visit their area and access sheep grazing land. We didn’t isolate oocysts or study seroprevalence in cats. Probably cats in some households are not infected at all and in this case presence of cats and their numbers is not directly related to seroprevalence. Similarly, the presences of wild felids viz. Leopard (*Panthera pardus*, Linnaeus, 1758), Serval (*Leptailurus serval*, Schreber, 1776), Cheetah (*Acinonyx jubatus*, Schreber, 1776) and African Lion (*Panthera leo*, Linnaeus, 1758), in the study areas did not affect the seroprevalence of toxoplasmosis.

The high seroprevalence in this study illustrates the probability of *T. gondii* transmission to consumers through mutton and lamb. A strong correlation between the seroprevalence of *T. gondii* in sheep and the risk of human infection has been reported [6,9,10,19,46]. The inadequate culinary hygiene, the inadequate meat freezing facility, “tasting” of the raw meat during the cooking process, and the widespread consumption of raw and undercooked meat in the study areas increases pressure of infection by *T. gondii* to humans.

## Conclusion

We conclude that *T. gondii* infection of sheep at flock and animal levels in the study districts is high and that altitude, sex, age, flock size and source of water are independent predictors of *T. gondii* seropositivity. Enforcing hygienic measures, the education of people and provision of rendering facilities to kill the bradyzoites in meat whenever possible are recommended. Further detailed studies to assess the impact of infections are warranted.

## Competing interests

The authors declare that they have no competing interests.

## Authors’ contributions

EZ conceived and designed the proposal, participated in the coordination and management of the study, collected, tested and analyzed the data and drafted the article. AA participated in sample collection, laboratory testing and drafting of article with inputs from MV, V di M, EC, JV and PD. TST, MV, V di M, EC, JV and PD participated in the study design and edition of article. GM made contribution in the data analysis and interpretation. All authors read and approved the final manuscript.
